# Persistent Post-endoscopic Retrograde Cholangiopancreatography Hiccups: An Unusual Presentation of Bile Reflux

**DOI:** 10.7759/cureus.39105

**Published:** 2023-05-16

**Authors:** Eli A Zaher, Parth Patel, Surendra Sigdel, George A Atia

**Affiliations:** 1 Department of Internal Medicine, Ascension St. Joseph Hospital, Chicago, USA; 2 Department of Gastroenterology and Hepatology, Ascension St. Joseph Hospital, Chicago, USA

**Keywords:** ercp, endoscopic retrograde cholangiopancreatography, hiccups, gastritis, bile reflux, bile reflux gastritis, acute pancreatitis, persistent hiccups, bile acid, endoscopy ercp

## Abstract

Bile reflux is a pathological retrograde flow of bile into the stomach that may lead to gastric overdistension and gastritis. It generally manifests as abdominal pain, nausea, vomiting, or heartburn. Hiccups have thus far not been described as part of its presentation. Here, we describe a case of excessive post-endoscopic retrograde cholangiopancreatography bile accumulation in the stomach that caused persistent hiccups requiring endoscopic suctioning.

## Introduction

Bile reflux occurs when secreted bile flows backward from the sphincter of Oddi into the stomach. It is commonly seen after procedures that involve manipulation of the biliary tree and pyloric sphincter. A retrospective cohort study found that its prevalence after endoscopic retrograde cholangiopancreatography (ERCP) is over 70%, with higher rates among diabetics and obese patients [1]. Cholecystectomy may impair duodenal motility from the unrestricted bile flow and lead to duodenogastric reflux [2,3]. Bile is an alkaline substance and can cause chemical injury to the normally acidic gastric mucosa. This manifests as abdominal pain, nausea, vomiting, or heartburn [1,3]. Hiccups have not been described in the literature as part of bile reflux symptoms. They occur from irritation of the reflex arc and are most commonly caused by rapid distention of the stomach [4]. We describe a case of a 56-year-old male who developed persistent hiccups and belching following ERCP for gallstone pancreatitis. Multiple medications and a cholecystectomy failed to resolve his symptoms. Diagnostic upper endoscopy aspirated 1.5 liters of bile reaching up to the middle esophagus.

## Case presentation

**Figure 1:**
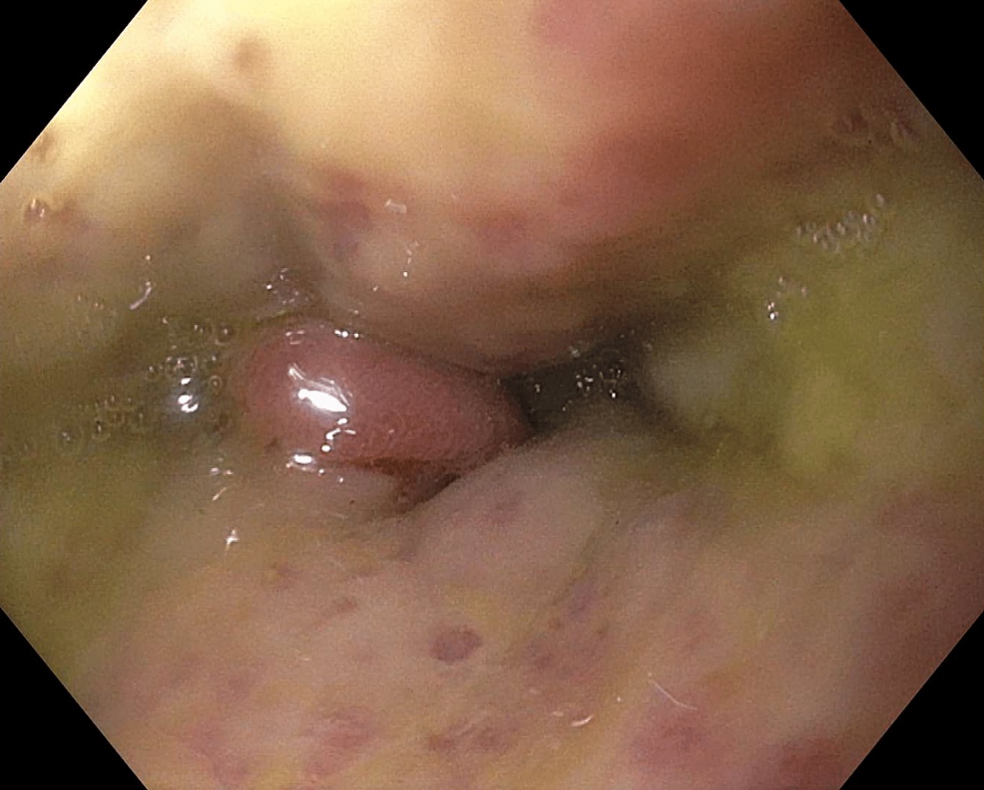
Pooling of bile above the gastroesophageal junction

A 56-year-old obese male with no past medical history presented to the emergency department with a one-day history of severe abdominal pain. CT of the abdomen demonstrated pancreatic head edema consistent with pancreatitis. Blood workup was remarkable for an elevated lipase of 1248 IU/L (normal range: 10-140 IU/L) and a white cell count of 26.1 k/mm cu (normal range: 4.5-11 k/mm cu), aspartate aminotransferase (AST) of 132 IU/L (normal range: 8-48 IU/L), alanine aminotransferase (ALT) of 191 IU/L (normal range: 7-55 IU/L), and total bilirubin of 10 mg/dL (normal range: 0.1-1.2 mg/dL). Ranson’s criteria score was 3. Magnetic resonance cholangiopancreatography (MRCP) showed choledocholithiasis adjacent to the porta hepatis with mild intrahepatic biliary ductal dilation. He underwent ERCP with biliary stenting without stone extraction. His liver function tests began downtrending and repeat lipase was normal. The following day, he developed belching and continuous hiccups. Symptomatic management was started with pantoprazole, cholestyramine, and prochlorperazine without effect. Escalation of treatment with the gradual addition of gabapentin, sucralfate, and baclofen was likewise unsuccessful. Despite the continued symptoms, he was tolerating food well. The hiccups remained unchanged even after a laparoscopic cholecystectomy on his fourth post-ERCP day. Diagnostic upper endoscopy revealed acute bile reflux esophagitis and a large volume of bile collection (Figure 1). A total of 1.5 liters of bilious fluid filling the middle and lower parts of the esophagus, body, and antrum of the stomach was aspirated. His hiccups resolved shortly after and he was discharged on cholestyramine and metoclopramide with a scheduled repeat ERCP in six weeks for stent removal and stone retrieval.

## Discussion

Bile reflux (or duodenogastric reflux) is the retrograde flow of bile into the stomach. It is caused by a combination of excessive bile secretion, reversed duodenal motility, and patency of the pyloric sphincter. Manipulation of the biliary tree (e.g., cholecystectomy, biliary sphincterotomy, and ERCP) causes uncontrolled bile secretion that overflows the duodenum and leads to dysmotility. Alkaline bile neutralizes the acidity of the stomach and turns it into a breeding ground for bacteria. Furthermore, bile acids and lysolecithin dissolve the fatty protective barrier of the gastric mucosa and expose it to hydrochloric acid, leading to gastritis [2,4-5]. It typically manifests as abdominal pain, dyspepsia, nausea, bilious vomiting, or heartburn [1,4]. Our patient lacked the above symptoms but had belching and persistent hiccups, which are not described in the literature. Bile reflux is diagnosed by directly visualizing bile accumulation proximally to the pyloric sphincter on endoscopy. Other less commonly used methods include testing gastric contents for bile acid and bilirubin, or biliary radionuclide scanning [4]. Available information on treatment is mostly targeted against bile reflux gastritis. It includes withdrawing medications that reduce gastrointestinal motility and a trial of a proton pump inhibitor (PPI), ursodeoxycholic acid, or sucralfate. Traditional Chinese medicine that includes weiyankang capsules and hydrotalcite has shown promise in a large meta-analysis [6]. Our patient was on both a PPI and sucralfate in addition to four other medications, without relief.

Hiccups are classified by duration and are considered persistent when over 48 hours and intractable when present for more than a month [7]. They are caused by involuntary contractions of the diaphragm and intercostal muscles leading to rapid air entry into the lungs with reflex closure of the glottis. Glottal closure is mediated by the recurrent laryngeal branch of the vagus nerve and is an important protective reflex that prevents significant hyperventilation in persistent hiccups [8]. The reflex arc of hiccups is complex. Afferent impulses are carried by the vagus, phrenic, and sympathetic nerve fibers (thoracic outflow T6-T12). Those are then processed in the spinal cord (C3-C6), medulla oblongata near the respiratory center, and hypothalamic reticular formation [8,9]. The efferent response comprises the phrenic and accessory nerves, which activate the diaphragm and intercostal muscles, respectively. An insult to any part of this reflex arc could trigger hiccups [8]. The most common cause of hiccups is rapid distension of the stomach, which normally has a capacity of 1.1 to 1.9 liters [10]. This usually happens from the rapid consumption of a large meal or carbonated drinks. Endoscopy for our patient aspirated 1.5 liters of bile reaching the middle esophagus. In addition to gastric overdistension, bile likely caused irritation of the stomach wall triggering peripheral afferent impulses via mechanosensitive receptors, which detect static force and nociception [11].

## Conclusions

While hiccups are mostly caused by overdistension of the stomach from meals and carbonated drinks, they could be caused by any manipulation of the hiccup reflex arc, biliary tree, or pyloric sphincter. Bile reflux is an unusual cause of hiccups but should be considered in post-ERCP patients, especially if persistent despite medical therapy. Management involves suctioning of the pooled bile via endoscopy.
